# The effect of educational intervention based on the PEN-3 model on breast cancer screening behaviors

**DOI:** 10.3389/fpubh.2023.1123888

**Published:** 2023-08-01

**Authors:** Elaheh Shoushtari-Moghaddam, Hossein Shahnazi, Akbar Hassanzadeh

**Affiliations:** ^1^Department of Health Education and Health Promotion, School of Health, Isfahan University of Medical Sciences, Isfahan, Iran; ^2^Department of Epidemiology and Biostatistics, School of Health, Isfahan University of Medical Sciences, Isfahan, Iran

**Keywords:** breast cancer, screening, PEN-3 model, education, teacher

## Abstract

**Background:**

Breast cancer is the most important malignancy and the main cause of cancer deaths among women worldwide. Breast cancer screening is an effective way to reduce breast cancer deaths.

**Objectives:**

The present study aimed to provide breast cancer screening behavior training for female teachers in Isfahan, Iran.

**Materials and methods:**

This randomized controlled trial included 120 teachers who have randomly divided into two groups (60 in the control group and 60 in the intervention group). The data collection tool was a PEN-3 model-based questionnaire. Four 90 min training sessions were held for the intervention group to modify perception and enablers. Breast self-examination (BSE), Clinical breast exam (CBE), and mammography (MMG) were investigated in both groups before and after 6 months after the last training using SPSS20 and appropriate statistical tests.

**Results:**

The frequency of BSE (*p* = 0.02), CBE (*p* = 0.04), and MMG (*p* = 0.01) in the intervention group was significantly higher than in the control group 6 months after training. The mean scores of perception and enablers were significantly higher in the intervention group than in the control group 3 and 6 months after training (*p* < 0.001). The logistic regression analysis indicated that perception factors were the strongest predictors of breast cancer screening behavior in teachers.

**Conclusion:**

Results of the present study indicated that using the PEN-3 model in the educational intervention was effective in improving breast cancer screening behavior.

## Introduction

Breast cancer is the most common cancer type in women and the leading cause of death in the world (1). The incidence of breast cancer is about 25 cases per 100,000 women in Iran (2), and it is especially 30 cases per 100,000 women in the central regions of the country (3). Most Iranian women with breast cancer are between the ages of 40 and 50, and most of them are at stage 2 in terms of levels of breast cancer (4). The burden of this disease is a challenge for the health economy in low- and middle-income countries such as Iran (5). Early detection of breast cancer is the most effective way to reduce mortality from the disease (6); thus, screening programs such as breast self-examination (BSE), clinical breast exam (CBE), and mammography (MMG) have been developed to detect early breast cancer (4). Unfortunately, studies conducted in Iran indicate that the adherence rate to the recommended guidelines for breast cancer screening among Iranian women is low (7, 8). The results of a systematic review on the early detection of breast cancer in Iran showed that only 21.9%, 15.8%, and 16.7% of Iranian women perform breast self-examination, clinical examination, and mammography, respectively (7). On the other hand, there is no specific program for breast cancer screening in Iran’s healthcare networks. Therefore, people should pay for mammography and other screening procedures at their own expense. Many women miss out on early detection and treatment of breast cancer due to a lack of information and awareness about the disease and its screening behaviors (9). For example, only 42.7% of women in Mosul, Iraq, have a good knowledge level (10). Therefore, there is a need for appropriate knowledge, perceptions, and beliefs in individuals to carry out screening programs (11). As in the study of Ghaffari et al., the effectiveness of educational interventions in increasing knowledge about cancer and screening behaviors was proven (12).

**Figure 1:**
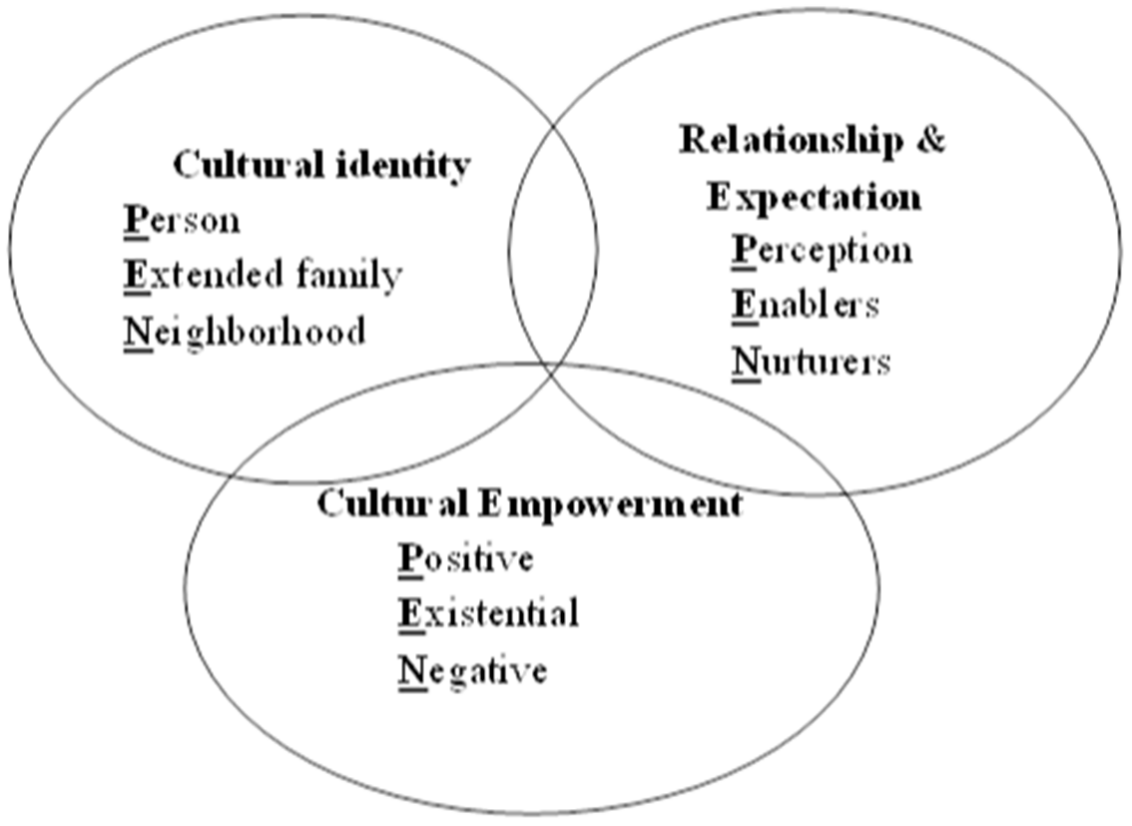
The PEN-3 model.

The first step in planning educational programs and effective health education is to select a health education model tailored to each event (13). Since many behaviors are influenced by society’s culture, the correct identification of the dominant culture is a prerequisite for the implementation of behavior change interventions. Several studies have highlighted the impact of culture-based educational interventions, all of which have led to increased breast cancer screening behavior (14, 15). The PEN-3 model is a health education model that helps us understand the role of society in strengthening positive behavior and enabling individuals to do that behavior (16). The cultural PEN-3 model includes three interrelated main dimensions, and each dimension has three factors that constitute the PEN abbreviation. The first dimension is cultural identity: (P) Individual: individuals will be sensitive and committed to engaging in the desired health behavior. (E) Extended family: it aims to bring together extended families and relatives. (N) Neighborhood: involving the neighborhood and society in the development of appropriate health behaviors while taking the culture of that neighborhood and society into account. The second dimension is relationships and expectations: (P) Perceptions are beliefs, values, and attitudes that can facilitate or prevent changes in behavior. (E) Enablers: the forces that can cause or prevent the occurrence of health-related behavior. (N) Neighborhood: involving the neighborhood and society in the development of appropriate health behaviors while taking into account their culture. Cultural empowerment is the third dimension. (P) Positive: beliefs and behaviors that help a person engage in healthy behavior. (E) Existential: these beliefs are neither good nor bad, but they exist in the person’s culture and should be taken into account; we should be aware of them. These beliefs are unique in every culture. (N) Negative: negative beliefs that impede good health behavior (Figure 1) (17).

Because teachers are the most important role models for students, they have been selected as the target group for the current study.

Two hypotheses were involved in this study: H1. The mean scores of perceptual factors and enablers within and between the intervention and control groups are different 3 and 6 months after educational intervention. H2. In the intervention group, each breast cancer screening behavior will be significantly more frequent 6 months after training.

Given the suggestion of using the PEN-3 model in educational interventions for breast cancer screening in descriptive studies in Iran (18, 19), the present study investigated the impact of educational intervention based on some dimensions of the PEN-3 model on breast cancer screening behavior among female teachers in Isfahan.

## Materials and methods

### Study design and participants

The present quasi-experimental study was conducted on 120 female teachers working at first-grade high schools in District 3 of the education department in Isfahan, Iran. Consent to participate in the study and not having a history of breast cancer were considered inclusion criteria. Failure to participate in training sessions, not being interested in continuing cooperation, and breast cancer diagnosis during the study were the exclusion criteria. The participants were divided into two groups (60 in the control group and 60 in the intervention group). Then, a pre-test was administered to both groups using the PEN-3 questionnaire. The intervention group received training based on PEN-3 model constructs, but the control group did not receive any education. The first author of the study was responsible for implementing educational sessions. The research team participated in designing educational content and strategies like role-playing and demonstrations. After that, the teachers were followed up and completed the post-test after 3 and 6 months (18).

### Sampling

Multi-stage cluster sampling was used in this study. One out of six districts of Isfahan’s education department and then 12 schools out of 24 state high schools were selected randomly using the drawing method. Among them, six schools were randomly considered to select individuals in the intervention group, and six schools were considered for selecting control group individuals using a table of random numbers. Ten teachers were selected voluntarily from each school via convenience sampling to participate in the research.

According to the equation of = $\frac{{\left(z_{1}+z_{2}\right)}^{2}\left(2s^{2}\right)}{d^{2}}$ , a sample size of at least 43 was obtained. Assuming a 20% drop in the sample size of each group, at least 53 people were chosen. In this equation, *z*_1_ is the confidence level of 95% equal to 1.96, *z*_2_ is the power factor of 80% equal to 0.84, *s* is an estimate of the standard deviation of each variable in both groups, and *d* is the minimum difference between each variable in both groups with a significant difference equal to 0.6 s (18).

Three and six months after the intervention, the post-test was administered in both the intervention and control groups to examine the effects of education on primary and secondary outcomes. The primary outcomes of the current research included the constructs of PEN-3 (perceptions and enabler factors), and the secondary outcome was breast cancer screening behavior in women. According to experts’ opinions, due to the time required to make an appointment for mammography and clinical examinations, 3 months of follow-up was not enough to perform breast cancer screening behaviors, so the behaviors were assessed only before and 6 months after training.

Based on the nature of the intervention in the present study, the instructor was not blinded to group assignment, but participants and the statistical investigator were blinded to group assignment.

### Data collection tool

The data collection tool was a PEN-3 model-based questionnaire with approved validity and reliability in a study by Naghibi et al. The validity of the questionnaire was theoretically determined using the content validity and consultation with 10 experts, and its reliability was obtained by Cronbach’s alpha test for each section of the questionnaire: screening function (*α* = 0.80), perception (*α* = 0.81), enablers (*α* = 0.78), and nurturers (*α* = 0.77) (20). In the present study, Cronbach’s alpha values were 0.86, 0.91, 0.80, and 0.78 for the screening function, perception, enablers, and nurturers, respectively.

The questionnaire consists of five sections. The first part contains 13 questions about personal and social characteristics; the second part contains six questions about women’s behavior in early diagnosis of breast cancer; the third part has 24 questions about perception factors such as “The risk of breast cancer increases with age; I feel that I am not able to perform breast self-examination.” The fourth part has 13 questions about enablers. Such as “Are the health care unit and doctor’s office available to receive information about breast self-examination and breast examination?; Is having health insurance effective for going to the doctor for breast examination and mammography?; Breast self-examination, clinical examination, and mammography are time-consuming?.” The fifth part has 13 questions about nurturers, such as “My husband encourages me to do breast self-examination, breast examination by specialist, and mammography; My family members (my mother, my sister, etc.) agree to perform breast self-examination, breast examination by specialists, and mammography; The advice of religious leaders lead to breast self-examination, breast examination by specialists, and mammography.” For scoring items of the questionnaire, a five-point Likert scale (totally agree = 5, agree = 4, no idea = 3, disagree = 2, and disagree = 1) with a score range of 24–120 was used. Questions in the “enablers and nurturers” section had Yes/No types. The scores ranged from 0–13 for the “enablers” section and 0–13 for the “nurturers” section. The questions in the behavior section had Yes/No and multiple-choice types. The frequency of behavior was considered for every question.

### Intervention

The questionnaires were first completed with samples from two groups, and then the intervention group members were trained. After 3 and 6 months, the questionnaires were re-completed by them. The training sessions included four sessions, each of which provided 90 min for teachers in the intervention group. The behavioral goals of the training sessions were developed based on the factor P (person) of the first dimension, second dimension (except Nurturers), and third dimension of the PEN-3 model, taking into account the positive and negative beliefs obtained from the previous study (18) to change and strengthen the perceptual and enabling factors that influence screening behaviors. Using the PEN-3 model, which addresses culture by considering the Positive and Negative aspects of people’s behaviors and beliefs, is useful for training, but due to time and performance constraints, we could not consider nurturers, which had a significant impact on screening behavior, as interventions to change behavior.

The first two sessions of training were conducted using lectures, brainstorming, question and answer, and group discussion based on perception to change and promote the teachers’ beliefs, values, and attitudes towards breast cancer screening behavior. The second two sessions were held through lectures, questions, and answers, group discussion, showing videos and photos, playing roles, and demonstrations based on enabling factors for empowering teachers to produce screening behavior. The intervention was as follows:

#### Training based on perceptions factors

***Awareness***: improved teachers’ awareness of breast cancer risk factors and screening methods and their timing using lecture and question-and-answer methods.

***Beliefs***: using brainstorming, teachers’ positive and negative beliefs about screening behavior were identified; and group discussions were conducted to eliminate false beliefs and create and maintain positive beliefs. For example, teachers explained that attention to women’s health is very important, and screening behavior is a sign of attention to female health, and breast cancer is like any other illness such as heart disease, in that both of them are preventable and are related to lifestyle and environmental factors, not fate and destiny. In addition, the participants also discussed their positive experiences with disease prevention and diagnosis methods for themselves and their relatives as a group.

***Attitude***: the goal was to change attitudes towards breast cancer screening methods through lectures, questions and answers, and group discussions. Using a group discussion, participants with a history of breast cancer screening behavior expressed their positive experience and also talked about the consequences of such behavior, such as the early diagnosis of any mass in the breast and the importance of self-examination.

#### Training based on enablers factors

The government centers that provide screening services in Isfahan were introduced for teachers.

The skills of breast self-examination were taught through a practical demonstration by the researcher, and then this behavior was dramatically played and screened by some learners. They also learned about a variety of breast cancer screening methods and how to perform a breast self-examination.

Regular planning for screening procedures was set up and made available to everyone by receiving feedback through the assistance of all participants in the training sessions, and they had been taught that the planning might change according to each individual’s circumstances. For example, mammography timing is different for risky and ordinary people.

At the end of the training sessions, participants were provided with a summary of the content in the form of a booklet. Also, 6 months after the training, the participants were followed up by sending training text messages.

### Analysis

SPSS (Version 20) software was used for data analysis. The independent *t*-test was used to test the differences between the model constructs at different times between the two groups. The repeated measures ANOVA was used to check the difference between the investigated variables at different times in each group, and logistic regression analysis was used to determine the predictive power of each perceptual and enabling factor regarding behavior. Frequency distribution, Chi-square, and Mann–Whitney tests were also used to examine individual and social characteristics.

### Ethical considerations

The present study was approved by the Ethics Committee of Isfahan University of Medical Sciences (Confirmation code: (IR.MUI.REC.1396.697)), and registered in Iran Registry Clinical Trials (IRCT) (code: IRCT20180516039690N1). After explaining the objectives of the study, participants completed the written consent forms and were assured of the confidentiality of their information. Furthermore, the participants were informed that they had the right to withdraw from the study at any time and were assured of the confidentiality of the study. In general, researchers have adhered to the Declaration of Helsinki (21). Moreover, after the study, training materials, such as booklets, were given to the control group.

## Results

The age range of teachers was 40 to 57 years in the control group, and 40 to 54 years in the intervention group. The independent *t*-test indicated that the mean age (*p* = 0.30), age of the first menstruation (*p* = 0.59), age of the first pregnancy (*p* = 0.20), and age of menopause (*p* = 0.37) were not significantly different between the two groups. The chi-square test indicated that the frequency distribution of history of breast disease in themselves (*p* = 0.61), and the frequency of history of breast disease in relatives (*p* = 0.49) did not differ significantly between the two groups (Table 1).

**Table 1:** Comparing participants’ demographic characteristics.

Variable		Intervention group *N* = 60	Control group *N* = 60	*p*-value
		Mean (Standard deviation)	Mean (Standard deviation)	
Age (years)		46.23 (3.9)	47.31 (4.2)	0.30^*^
Age of first menstruation (years)		14.27 (1.5)	14.02 (1.4)	0.59^*^
Age of first pregnancy (years)		24.94 (3.9)	23.92 (3.7)	0.20^*^
Number of children		2.03 (0.6)	2.02 (0.7)	0.99^*^
Age of menopause (years)		47.77 (4.7)	48.70 (3.01)	0.37^*^
Height (centimeters)		162.17 (7.2)	161.54 (5.1)	0.64^*^
Weight (kg)		68.55 (10.2)	65.71 (11.5)	0.19^*^
		Number (Percentage)	Number (Percentage)	
Marital status	Single	3 (5.08)	5 (8.47)	0.58^**^
	Married	55 (91.54)	52 (86.46)	
	Divorced	2 (3.38)	2 (3.38)	
	Died husband	0 (0)	1 (1.69)	
Husband’s job	Employed	41 (74.48)	37 (71.18)	0.92^**^
	Unemployed	2 (3.62)	2 (3.81)	
	Retired	12 (21.90)	13 (25.01)	
History of breast disease in relatives	No	41 (68.15)	41 (68.15)	0.94^**^
	First degree relatives	6 (10.23)	5 (8.37)	
	Second degree relatives	13 (21.62)	14 (23.48)	
Hormone therapy during menopause		2 (3.34)	4 (6.72)	0.34^**^
History of breast disease		10 (16.73)	8 (13.33)	0.61^**^
Education	Associate degree	3 (5.00)	7 (11.66)	0.27^**^
	Bachelor	41 (68.32)	40 (66.62)	
	Master	16 (26.68)	13 (21.72)	
Spouse education	High school diploma	6 (10.90)	6 (11.56)	0.64^**^
	Associate Degree	7 (12.72)	8 (15.38)	
	Bachelor	27 (49.09)	19 (36.53)	
	Master	15 (27.29)	19 (36.53)	

*Independent *t*-test and **Chi-square test.

The mean and standard deviation of nurturers in the intervention and control groups were 65.6 ± 21.5 and 62.8 ± 24.6 respectively, and the independent *t*-test indicated that there was no significant difference between the two groups in the mean scores of nurturers (*p* = 0.51). The most effective nurturers in screening behavior among participants included encouraging family members with 86.7% and encouraging friends with 80%.

The chi-square test indicated that the frequency of breast self-examination (*p* = 0.77), frequency of clinical breast exam (*p* = 0.85), and frequency of mammography (*p* = 1) were not significantly different between the two groups before the intervention, but they were significantly higher in the intervention group than the control group 6 months after training, with *p*-values equal to *p* = 0.02, *p* = 0.04, and *p* = 0.1, respectively (Table 2).

**Table 2:** Comparing the frequency distribution of breast cancer screening behavior between groups before and 6 months after the intervention.

Behavior	Time	Intervention group *N* = 60		Control group *N* = 60		*p*-value^**^
		Number	Percentage	Number	Percentage	
Breast self-examination (BSE)	Before intervention	38	63.33	40	66.66	0.70
	6 months after intervention	49	81.66	39	65.00	0.02*
Clinical breast exam (CBE)	Before intervention	37	61.66	36	60.00	0.85
	6 months after intervention	45	75.00	37	66.66	0.04*
Mammography (MMG)	Before intervention	29	48.33	29	48.33	1
	6 months after intervention	41	68.33	28	46.66	0.01*

*Significant *p* ≤ 0.05 and **Chi-square test.

The Mann–Whitney U test indicated that there was no significant difference between both groups in terms of time intervals of breast self-examination before training (*p* = 0.80). However, 6 months after training, breast self-examination was better in the intervention group than in the control group (*p* < 0.001). In addition, the Wilcoxon test showed that there was no significant difference between the breast self-examination interval in the control group before and 6 months after the intervention (*p* = 0.91), but a significant difference was observed in the intervention group between the two times (*p* < 0.001) (Table 3).

**Table 3:** Comparing frequency distribution of breast self-examination interval before and 6 months after the intervention between and within groups.

Time		Intervention group *N* = 60		Control group *N* = 60		*p*-value *
		Number	Percentage	Number	Percentage	
Before intervention	Once a month	9	15.00	9	15.00	0.80
	Every six months	5	8.33	6	10.00	
	Once a year	4	6.66	3	5.00	
	Sometimes	20	33.34	22	36.67	
	Never	22	36.67	20	33.34	
6 months after intervention	Once a month	32	53.92	6	10.00	<0.001***
	Every six months	4	6.66	6	10.00	
	Once a year	2	3.33	5	8.33	
	Sometimes	11	24.42	22	36.67	
	Never	7	11.67	17	28.33	
*p*-value**		<0.001***		0.91		–

*Mann–Whitney, **Wilcoxon signed-rank test, and ***Significant *p* ≤ 0.01.

Before the intervention, 91 (75.8%) out of 120 participants had undergone at least one breast cancer screening behavior (BSE, CBE by a physician or medical staff, and MMG).

The independent *t*-test indicated that mean scores of perception factors (*p* = 0.08) and enablers (*p* = 0.30) had no significant difference between the two groups before the intervention. The analysis of covariance modified scores of perception factors and enablers in two groups before the intervention and indicated that mean scores of perception factors and enablers were significantly higher in the intervention than in the control group 3 and 6 months after training (*p* < 0.001) (Table 4). The LSD *post hoc* test showed that the mean score of perception factors and enablers 3 and 6 months after the intervention was significantly higher than before the intervention (*p* < 0.001). Also, the mean score of perceptual factors (*p* < 0.001) and enablers (*p* < 0.008) in the intervention group six months after the intervention was significantly lower than three months after the intervention.

**Table 4:** Comparing mean scores of perception and enabler factors (based on 100) between and within groups before intervention, 3 and 6 months after the intervention.

Time		Intervention group *N* = 60		Control group *N* = 60		*p*-value
		Mean	Standard deviation	Mean	Standard deviation	
Perception factors	Before intervention	71.02	10.8	74.47	9.8	0.08*
	3 months after the intervention	89.42	6.2	74.34	8.9	<0.001**
	6 months after the intervention	86.64	6.7	73.49	9.2	<0.001**
Test of significance***		*F* = 134.11, *p* = <0.001***		*F* = 0.31, *p* = 0.74		–
Enablers	Before intervention	63.11	21.2	66.9	19.57	*p* = 0.30*
	3 months after the intervention	88.95	12.4	68.7	19.42	*p* = <0.001**
	6 months after intervention	81.53	24.7	64.4	24.44	*p* = <0.001**
*p*-value***		<0.001***		0.14		–

*Independent *t*-test, **ANCOVA, and ***Repeated measures ANOVA.

## Discussion

This study looked into the effect of an educational intervention based on some dimensions of the PEN-3 model on breast cancer screening behaviors among female teachers in Isfahan. Results indicated that 91 out of 120 participants (75.8%) had at least one of the breast cancer screening behaviors (BSE, CBE by physician or medical staff, and MMG) before the intervention. However, the above-mentioned behaviors of the participants increased significantly after the intervention. In the present study, there were no significant differences between the two groups in terms of the time interval for breast self-examination, the frequency of mammography, or the clinical exam before the intervention. However, the behavior was better in the intervention group than in the control group after 6 months of the intervention. The results indicated that training based on the PEN-3 model significantly affected behavior change. In a study by Bayik Temel et al., after the intervention, there was a significant increase in performing mammography and self-examination and knowing the appropriate time, indicating the impact of training. Half of the teachers did not perform any self-examination, and they had not even undergone mammography before receiving training in this study (22). Through a panel discussion in a PowerPoint presentation, Lee et al. studied the impact of the intervention on increasing breast cancer screening in Korean–American couples, and the effects of the intervention became maximal after 6 months. Their results indicated that the intervention group performed mammography twice as often as the control group (Eunice (23)). Some other studies indicate that the intervention had a large effect on mammogram completion post-intervention (14, 15). Therefore, it can be concluded that paying attention to culture is very effective in training healthy behaviors, which can achieve by using the PEN-3 model.

The results of this study showed that the mean score of perception factors in the intervention group at 3 and 6 months after the intervention was significantly higher than those in the control group. The perception factors of this study included increased knowledge about risk factors, screening methods, and their timing, as well as the impact of timely breast cancer diagnosis and treatment. The group discussion and messages sent to participants were very useful in this regard. According to a study by Bryan et al., there was a significant change in participants’ attitudes toward performing breast cancer screening behaviors after educational intervention (24). In a study by Ghaffari et al., there was a significant increase in participants’ knowledge about breast cancer screening after the intervention (12). In a study by Temel et al., the increased knowledge of risk factors and screening methods had a positive effect on screening behavior (22). These results indicated that having knowledge and a positive attitude towards behavior is important for changing it in a positive way [Eunice (23)].

According to this study, the mean score of enablers in the intervention group was significantly higher than in the control group. The teachers were introduced to the method of screening behavior between 3 and 6 months after the intervention, most likely through education, and breast self-examination can be performed through role-playing and demonstration. In several studies in Iran, there was a significant increase in the rates of perceived benefits and barriers to performing screening behaviors after the intervention compared to before the intervention (12, 25). In another study, participants mentioned several factors, such as the lack of knowledge about performing breast self-examination and having no regular monthly program for doing it, as well as shame and embarrassment and spending a lot of time on a clinical examination and breast self-examination, as existing barriers. The obstacle was partially resolved, and the behavior increased significantly after the intervention (6). Results of a study by Ka’opua also indicated that participants considered mammography useful for health and that those who performed screening might leave or postpone it due to fear of mass diagnosis, concern about costs, and being unsure about free services in the screening program (26). In a study by Cohen et al., participants in the intervention group reported perceiving fewer barriers (e.g., religious barriers and a significant effect of time was obvious for social barriers) after the intervention compared with the control group (14). These results showed the significance of resources such as time and finances that should be considered for educating on disease prevention.

Nurturers are among the determinants of screening behavior. In the present study, family members’ encouragement, which was positive nurture, had the greatest impact on screening behavior. Sheppard et al. found that family members played key roles in deciding on breast cancer treatment (27). According to Dong et al., the most important nurturers of breast cancer screening behavior were family members’ awareness and encouragement (28). The support of the family, especially the spouse, is a very important factor for women in performing screening behaviors (23).

Results of the present study indicated that scores of perception factors were significant predictors for performing or not-performing breast cancer screening behavior. Baron Epel considered belief in fate and fear of breast cancer diagnosis, which were among perception factors, as predictors of mammography among the number of participants (29). According to Tavafian et al., the understanding of lower barriers and higher self-efficacy as enablers was the most important predictor of breast cancer screening behavior. In fact, those who had confidence in their ability to perform the breast self-examination showed more such behavior (30). The most important predictors of a study by Tolma et al. were the encouragement and recommendation by the physician to perform screening behavior and a better understanding of screening methods (31). Knowledge, which is a perceptual factor, is considered a necessity for behavior change, but while increasing knowledge, other factors, such as enabling factors and nurturing factors, should also be considered to change behavior and achieve better results.

## Conclusion

Based on the obtained results, the use of the PEN-3 model in educational interventions is highly effective in promoting breast cancer prevention behavior because this model not only focuses on individual aspects but also emphasizes the influence of the environment and surrounding people. By using this model to consider positive and negative beliefs, all effective factors, including perceptions, enabling factors, and nurturers, can be applied to educational interventions. It is also important to educate teachers as people who influence society, become role models for students, and play a role in the transfer of knowledge and awareness. Therefore, it is suggested that in future studies, the teachings received by teachers be transferred to their students, and the results be measured in students, too.

### Strengths and limitations

The strength of the present study is that the design of the educational intervention was based on the PEN-3 model as well as on the follow-up of the behavior of teachers 6 months after the educational intervention.

The limitations of the present study were as follows: due to time and performance constraints, we could not consider nurturers for intervention, which had a significant impact on screening behavior as interventions to change the behavior. Furthermore, the first dimension of the PEN-3 model, indicating the impact of neighborhood and extended family on behavior implementation or change, was not utilized in the present study. Hence, it is suggested to use all three dimensions of the PEN-3 model to perform or change breast cancer screening behavior in future studies. This study was conducted among teachers from a specific area in Iran, and the results may not be generalizable to other populations, so conducting a study in another population is recommended. Another limitation of this study was that it was designed in a quasi-experimental way. If it were a clinical trial study, it would be possible to draw better and more accurate conclusions by following the principles of CONSORT.

## Data availability statement

The original contributions presented in the study are included in the article/supplementary material, further inquiries can be directed to the corresponding author.

## Ethics statement

The present study was confirmed by the Ethics Committee of Isfahan University of Medical Sciences (Confirmation code: (IR.MUI.REC.1396.697)), and registered in Iran Registry Clinical Trials (IRCT) (code: IRCT20180516039690N1). The patients/participants provided their written informed consent to participate in this study.

## Author contributions

HS and ES-M initially conceived and designed the study, wrote the paper and made revisions, and reviewing the manuscript critically. AH conducted the analysis. All authors contributed to the article and approved the submitted version.

## Conflict of interest

The authors declare that the research was conducted in the absence of any commercial or financial relationships that could be construed as a potential conflict of interest.

## Publisher’s note

All claims expressed in this article are solely those of the authors and do not necessarily represent those of their affiliated organizations, or those of the publisher, the editors and the reviewers. Any product that may be evaluated in this article, or claim that may be made by its manufacturer, is not guaranteed or endorsed by the publisher.
